# The Gene Ontology knowledgebase in 2023

**DOI:** 10.1093/genetics/iyad031

**Published:** 2023-03-03

**Authors:** The Gene Ontology Consortium, Suzi A Aleksander, James Balhoff, Seth Carbon, J Michael Cherry, Harold J Drabkin, Dustin Ebert, Marc Feuermann, Pascale Gaudet, Nomi L Harris, David P Hill, Raymond Lee, Huaiyu Mi, Sierra Moxon, Christopher J Mungall, Anushya Muruganugan, Tremayne Mushayahama, Paul W Sternberg, Paul D Thomas, Kimberly Van Auken, Jolene Ramsey, Deborah A Siegele, Rex L Chisholm, Petra Fey, Maria Cristina Aspromonte, Maria Victoria Nugnes, Federica Quaglia, Silvio Tosatto, Michelle Giglio, Suvarna Nadendla, Giulia Antonazzo, Helen Attrill, Gil dos Santos, Steven Marygold, Victor Strelets, Christopher J Tabone, Jim Thurmond, Pinglei Zhou, Saadullah H Ahmed, Praoparn Asanitthong, Diana Luna Buitrago, Meltem N Erdol, Matthew C Gage, Mohamed Ali Kadhum, Kan Yan Chloe Li, Miao Long, Aleksandra Michalak, Angeline Pesala, Armalya Pritazahra, Shirin C C Saverimuttu, Renzhi Su, Kate E Thurlow, Ruth C Lovering, Colin Logie, Snezhana Oliferenko, Judith Blake, Karen Christie, Lori Corbani, Mary E Dolan, Harold J Drabkin, David P Hill, Li Ni, Dmitry Sitnikov, Cynthia Smith, Alayne Cuzick, James Seager, Laurel Cooper, Justin Elser, Pankaj Jaiswal, Parul Gupta, Pankaj Jaiswal, Sushma Naithani, Manuel Lera-Ramirez, Kim Rutherford, Valerie Wood, Jeffrey L De Pons, Melinda R Dwinell, G Thomas Hayman, Mary L Kaldunski, Anne E Kwitek, Stanley J F Laulederkind, Marek A Tutaj, Mahima Vedi, Shur-Jen Wang, Peter D’Eustachio, Lucila Aimo, Kristian Axelsen, Alan Bridge, Nevila Hyka-Nouspikel, Anne Morgat, Suzi A Aleksander, J Michael Cherry, Stacia R Engel, Kalpana Karra, Stuart R Miyasato, Robert S Nash, Marek S Skrzypek, Shuai Weng, Edith D Wong, Erika Bakker, Tanya Z Berardini, Leonore Reiser, Andrea Auchincloss, Kristian Axelsen, Ghislaine Argoud-Puy, Marie-Claude Blatter, Emmanuel Boutet, Lionel Breuza, Alan Bridge, Cristina Casals-Casas, Elisabeth Coudert, Anne Estreicher, Maria Livia Famiglietti, Marc Feuermann, Arnaud Gos, Nadine Gruaz-Gumowski, Chantal Hulo, Nevila Hyka-Nouspikel, Florence Jungo, Philippe Le Mercier, Damien Lieberherr, Patrick Masson, Anne Morgat, Ivo Pedruzzi, Lucille Pourcel, Sylvain Poux, Catherine Rivoire, Shyamala Sundaram, Alex Bateman, Emily Bowler-Barnett, Hema Bye-A-Jee, Paul Denny, Alexandr Ignatchenko, Rizwan Ishtiaq, Antonia Lock, Yvonne Lussi, Michele Magrane, Maria J Martin, Sandra Orchard, Pedro Raposo, Elena Speretta, Nidhi Tyagi, Kate Warner, Rossana Zaru, Alexander D Diehl, Raymond Lee, Juancarlos Chan, Stavros Diamantakis, Daniela Raciti, Magdalena Zarowiecki, Malcolm Fisher, Christina James-Zorn, Virgilio Ponferrada, Aaron Zorn, Sridhar Ramachandran, Leyla Ruzicka, Monte Westerfield, Suzi A Aleksander, James Balhoff, Seth Carbon, J Michael Cherry, Harold J Drabkin, Dustin Ebert, Marc Feuermann, Pascale Gaudet, Nomi L Harris, David P Hill, Raymond Lee, Huaiyu Mi, Sierra Moxon, Christopher J Mungall, Anushya Muruganugan, Tremayne Mushayahama, Paul W Sternberg, Paul D Thomas, Kimberly Van Auken, Jolene Ramsey, Deborah A Siegele, Rex L Chisholm, Petra Fey, Maria Cristina Aspromonte, Maria Victoria Nugnes, Federica Quaglia, Silvio Tosatto, Michelle Giglio, Suvarna Nadendla, Giulia Antonazzo, Helen Attrill, Gil dos Santos, Steven Marygold, Victor Strelets, Christopher J Tabone, Jim Thurmond, Pinglei Zhou, Saadullah H Ahmed, Praoparn Asanitthong, Diana Luna Buitrago, Meltem N Erdol, Matthew C Gage, Mohamed Ali Kadhum, Kan Yan Chloe Li, Miao Long, Aleksandra Michalak, Angeline Pesala, Armalya Pritazahra, Shirin C C Saverimuttu, Renzhi Su, Kate E Thurlow, Ruth C Lovering, Colin Logie, Snezhana Oliferenko, Judith Blake, Karen Christie, Lori Corbani, Mary E Dolan, Harold J Drabkin, David P Hill, Li Ni, Dmitry Sitnikov, Cynthia Smith, Alayne Cuzick, James Seager, Laurel Cooper, Justin Elser, Pankaj Jaiswal, Parul Gupta, Pankaj Jaiswal, Sushma Naithani, Manuel Lera-Ramirez, Kim Rutherford, Valerie Wood, Jeffrey L De Pons, Melinda R Dwinell, G Thomas Hayman, Mary L Kaldunski, Anne E Kwitek, Stanley J F Laulederkind, Marek A Tutaj, Mahima Vedi, Shur-Jen Wang, Peter D’Eustachio, Lucila Aimo, Kristian Axelsen, Alan Bridge, Nevila Hyka-Nouspikel, Anne Morgat, Suzi A Aleksander, J Michael Cherry, Stacia R Engel, Kalpana Karra, Stuart R Miyasato, Robert S Nash, Marek S Skrzypek, Shuai Weng, Edith D Wong, Erika Bakker, Tanya Z Berardini, Leonore Reiser, Andrea Auchincloss, Kristian Axelsen, Ghislaine Argoud-Puy, Marie-Claude Blatter, Emmanuel Boutet, Lionel Breuza, Alan Bridge, Cristina Casals-Casas, Elisabeth Coudert, Anne Estreicher, Maria Livia Famiglietti, Marc Feuermann, Arnaud Gos, Nadine Gruaz-Gumowski, Chantal Hulo, Nevila Hyka-Nouspikel, Florence Jungo, Philippe Le Mercier, Damien Lieberherr, Patrick Masson, Anne Morgat, Ivo Pedruzzi, Lucille Pourcel, Sylvain Poux, Catherine Rivoire, Shyamala Sundaram, Alex Bateman, Emily Bowler-Barnett, Hema Bye-A-Jee, Paul Denny, Alexandr Ignatchenko, Rizwan Ishtiaq, Antonia Lock, Yvonne Lussi, Michele Magrane, Maria J Martin, Sandra Orchard, Pedro Raposo, Elena Speretta, Nidhi Tyagi, Kate Warner, Rossana Zaru, Alexander D Diehl, Raymond Lee, Juancarlos Chan, Stavros Diamantakis, Daniela Raciti, Magdalena Zarowiecki, Malcolm Fisher, Christina James-Zorn, Virgilio Ponferrada, Aaron Zorn, Sridhar Ramachandran, Leyla Ruzicka, Monte Westerfield

**Keywords:** Gene Ontology, gene annotation, gene function, knowledgebase, knowledge graphs

## Abstract

The Gene Ontology (GO) knowledgebase (http://geneontology.org) is a comprehensive resource concerning the functions of genes and gene products (proteins and noncoding RNAs). GO annotations cover genes from organisms across the tree of life as well as viruses, though most gene function knowledge currently derives from experiments carried out in a relatively small number of model organisms. Here, we provide an updated overview of the GO knowledgebase, as well as the efforts of the broad, international consortium of scientists that develops, maintains, and updates the GO knowledgebase. The GO knowledgebase consists of three components: (1) the GO—a computational knowledge structure describing the functional characteristics of genes; (2) GO annotations—evidence-supported statements asserting that a specific gene product has a particular functional characteristic; and (3) GO Causal Activity Models (GO-CAMs)—mechanistic models of molecular “pathways” (GO biological processes) created by linking multiple GO annotations using defined relations. Each of these components is continually expanded, revised, and updated in response to newly published discoveries and receives extensive QA checks, reviews, and user feedback. For each of these components, we provide a description of the current contents, recent developments to keep the knowledgebase up to date with new discoveries, and guidance on how users can best make use of the data that we provide. We conclude with future directions for the project.

## Introduction

Genes encode gene products, often proteins but also noncoding RNA molecules (ncRNAs), that perform functions at the molecular, cellular, and organismal levels. The Gene Ontology (GO) knowledgebase provides a comprehensive, structured, computer-accessible representation of gene function, for genes from any cellular organism or virus. The GO knowledgebase has become a critical component of life science research, supporting analysis of large-scale experiments and biological systems (Duck *et al.* 2016). It is designed to make expert knowledge of gene function accessible for bench scientists as well as computational analyses. The basic model underlying GO is the “molecular biology paradigm” (Ashburner *et al.* 2000; Thomas 2017), in which there are three types (aspects) of functional characteristics used to describe gene function:

- Molecular function (MF): the activities performed by a gene product at the molecular level
- Cellular component (CC): the locations, relative to cellular structures, where MFs are performed
- Biological process (BP): a “biological program” comprising molecular activities acting in concert to achieve a particular outcome; this program can be at the cellular level or at the organism level of multicellular organisms.

**Fig. 1.**
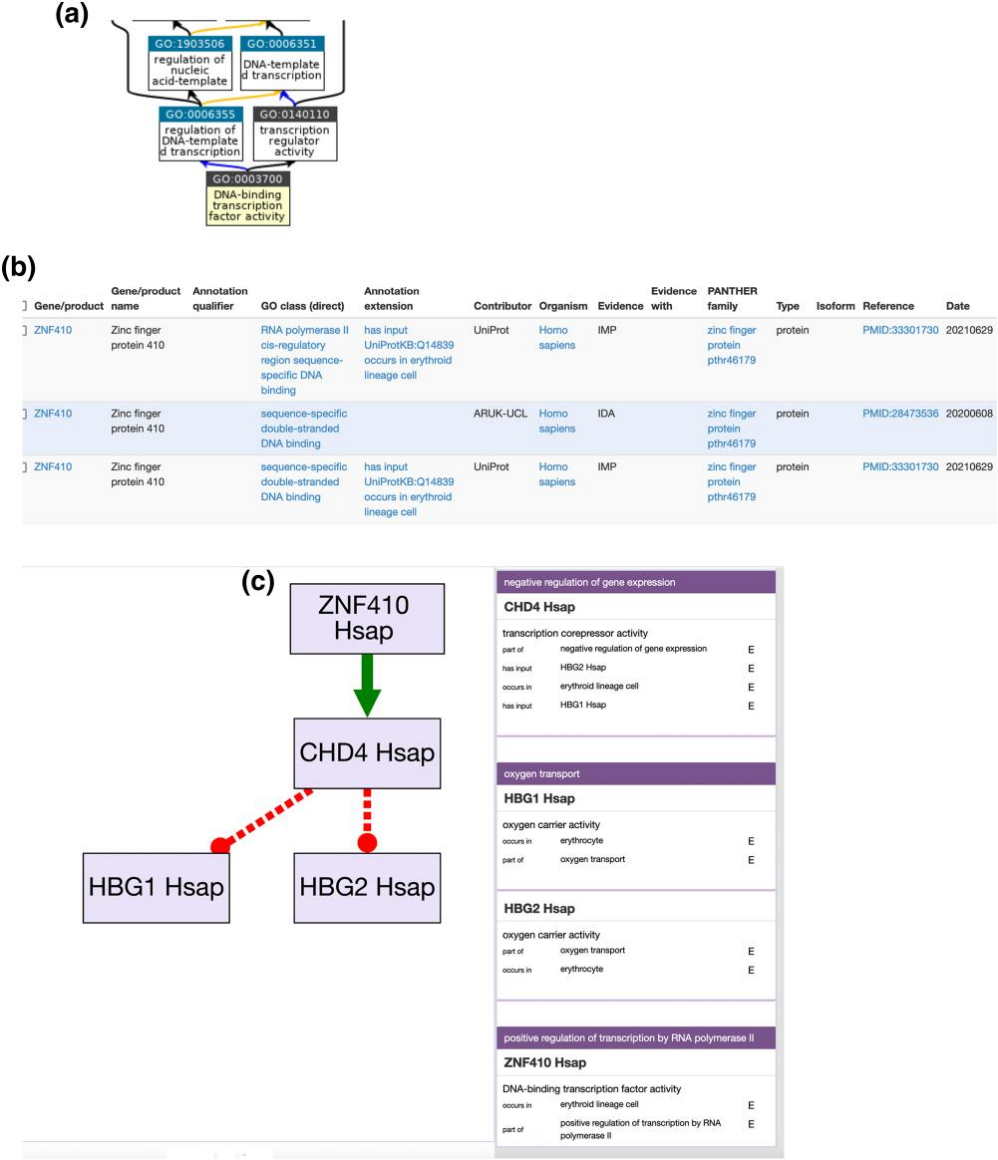
Examples of the three components of the GO knowledgebase. a) The GO ontology consists of terms, e.g. *DNA binding transcription factor activity*, and relationships between the terms (arrows; black = *is a*, blue = *part of*, and orange = *regulates*). b) GO annotations associate a specific gene product (here, human ZNF410) with GO terms asserting its functional aspects (“GO Class” column, e.g. sequence-specific double-stranded DNA binding) and the evidence for each assertion with its traceable source (“Evidence” and “Reference” columns). c) The GO-CAM model combines individual GO annotations into a model, in this case a very simple model describing how human ZNF410 acts as a transcription factor to positively regulate (denoted by the arrow) transcription of the *CHD4* gene, which in turn acts as a corepressor to repress (denoted by dashed lines) transcription of fetal hemoglobin genes (*HBG1* and *HBG2*) in erythroid lineage cells. In this view, each box in the GO-CAM is labeled with the gene product and species abbreviation for simplicity.

The GO knowledgebase consists of three components: the GO, GO annotations, and GO Causal Activity models (GO-CAMs) (Fig. 1). The GO (Fig. 1a) structures our current knowledge of the types of functional characteristics that a gene product may possess into a connected graph-based representation. Each ontology term (called “class” in the field of ontologies) represents a functional characteristic that can be attributed to a gene product. Terms can have relationships between them, such as one term being more specific than another term (also called “subclass”), e.g. *DNA-binding transcription factor activity* is a subclass of *transcription regulator activity*. A GO annotation (Fig. 1b) is an association between a specific gene (or gene product) and a GO term and should be interpreted as a statement that the specified gene product possesses the specified functional characteristic represented by the GO term. Each GO annotation includes the evidence upon which it is based. Because each GO annotation covers only a single characteristic of gene function, multiple GO annotations are generally required to completely describe the function of a gene product. GO-CAMs (Fig. 1c) link multiple GO annotations together to create models of BPs by (1) connecting the activities of more than one gene product together into causal networks and (2) allowing the specification of the biological context (e.g. cell type and tissue type) in which the activities occur.

## The GO knowledgebase is large and dynamic

For applications that use the components of the GO knowledgebase, it is crucial that the ontology and associated annotations represent the current state of knowledge and are not just an archive of all public data. Therefore, all aspects of the GO knowledgebase are dynamic (ontology, annotations, GO-CAMs, links to external ontologies, etc.), and citable, versioned updates are released on a monthly basis. Below, we describe each component of the knowledgebase, focusing on recent changes made to improve the resource during the past two years. Statistics and descriptions given here are based on the GO release 2022–11–03 (http://release.geneontology.org/2022-11-03, doi:10.5281/zenodo.7407024).

### Ontology

The ontology component of the GO knowledgebase consists of the terms used to describe functional characteristics of gene products, which are linked together by relations into a labeled directed acyclic graph (like a hierarchy but with multiple parentages allowed). It also includes term definitions, synonyms, and relations to terms from external ontologies. The GO is available in different editions, including (1) the “basic” edition, which includes only core relationship types; (2) the core ontology, including additional relationship types; and (3) the “go-plus” edition which also includes relationships to terms in other ontologies. These editions are explained on the GO downloads page http://geneontology.org/docs/download-ontology/. The ontology contains 43,303 terms (Table 2), linked together by 88,099 relationships in the basic edition. When relationships to external terms are included, there are 121,698 relationships; release statistics can be viewed at http://geneontology.org/stats.

The GO is subject to constant review and revision to most accurately model the current biological knowledge. Revision of the ontology includes the addition or obsoletion of terms and reorganization of the relationship structure. New GO terms are added to represent concepts previously missing from the GO in response to published findings, or when a branch of GO is revised. Terms may be obsoleted when unused or inconsistently used in annotation, when they are redundant with other terms, or during revision of specific branches of the ontology.

Most of the revisions in the structure of GO are in response to advances in biological knowledge, as well as improvements in the precision of newer experimental approaches. In addition, because many branches of the ontology have grown organically in a bottom-up fashion by accumulating specific individual term requests, we also perform systematic review aimed at improving consistency and clarity while reducing redundancy. Additional revisions are initiated by internal review, and consistency and quality assurance checks. Revisions are also made following feedback from users. Whenever possible, changes are performed in collaboration with expert biocurators or domain specialists; recent examples include blood–brain barrier-related functions (Saverimuttu *et al.* 2021) and transcription factors (Gaudet *et al.* 2021).

At each release, we track all changes and report on our website the number of added, obsoleted, and merged terms in the ontology. Table 1 shows the number of GO terms added and removed (merged or obsoleted) over the past two-year period, for each aspect of GO. In the MF and CC aspects of the ontology, term creation versus term obsoletion have approximately balanced each other, such that the number of terms in these two branches has remained roughly constant. The most significant changes have been in the BP aspect of GO, with a net decrease of over 800 terms.

**Table 1.** Changes to GO terms in the past two-year period. The ontology has undergone substantial revision and improvement, with nearly 2,000 terms added or removed.

GO aspect	Total number of terms	Added terms	Obsoleted terms	Merged terms*^a^*
Molecular function	11,271	315	65	143
Cellular component	4,039	34	19	162
Biological process	27,993	217	782	254

Also includes obsoleted terms that have been replaced by another term.

Many of these revisions result from global reviews of the ontology to address clear inconsistencies in usage and changes in annotation practices. Terms that have been removed from the ontology over the last two years fall into several different categories, including the following:

- **Terms that correspond to phenotypes** and for which the understanding of the process was previously too incomplete to annotate to a different term. Examples include the following: *regulation of spindle density* (GO:0090225) and *age-dependent general metabolic decline* (GO:0007571).
- Terms that are combinations of multiple GO terms that **can now be represented more precisely using GO-CAM models**. Examples are *chromatin remodeling in response to cation stress* (GO:0043156) and *regulation of cyclin-dependent protein serine/threonine kinase activity involved in G2/M transition of the mitotic cell cycle* (GO:0031660).
- **Revisions based on updated knowledge**, either by GO editors, by authoritative databases, or in the literature. For example, alpha-taxilin (UniProt:P40222) was originally thought to be the high-molecular weight interleukin-14 (Ambrus *et al.* 1993); an erratum was later published (Ambrus *et al.* 1996) indicating that the open reading frame had been incorrectly predicted. Hence, all terms mentioning interleukin-14 have been obsoleted [*interleukin-14 binding* (GO:0019974), *interleukin-14 production* (GO:0032617), and 5 other terms]. GO terms created from articles that have been retracted and for which no other supporting evidence exists are obsoleted, for example *CDP-acylglycerol O-arachidonoyltransferase activity* (GO:0047193) (Thompson and Zuk 1983). Some terms have been obsoleted from authoritative databases, for example EC:1.3.1.59 was removed from the Enzyme Commission database, and the corresponding GO term *1,6-dihydroxy-5-methylcyclohexa-2,4-dienecarboxylate dehydrogenase activity* (GO:0018512) was obsoleted in GO.
- **Single step reactions in the BP aspect of the ontology:** there were many instances in the GO where a MF could be represented as both a MF and a BP, for example “histone kinase activity” and “histone phosphorylation.” This was useful when fewer activities were characterized at the molecular level, and the best level of resolution for many experiments was that the gene has some uncharacterized role that led to histone phosphorylation, for example. However, with increasingly detailed molecular data, the redundancy between MF and BP annotations became unnecessary and the value of having a similar term in both aspects of the ontology led to inconsistency. This is an ongoing project and many BP terms still need to be obsoleted for this reason.
- **Terms that refer to more than one ontological aspect**: *ubiquinone biosynthetic process monooxygenase activity* (GO:0015997) included a BP within a MF; *MAP kinase phosphatase activity involved in regulation of innate immune response* (GO:0038078) included a MF within a BP; and *histone deacetylation at centromere* (GO:0031059) represented all three aspects: a MF in the BP branch of the ontology (histone acetylation) that also included CC information (centromere).
- **Misclassified terms**, for example *urea homeostasis* (GO:0097274) and *creatinine homeostasis* (GO:0097273): while these compounds are important medical biomarkers, the normal process that they measure is proper renal function; therefore, these terms have been obsoleted. Annotations have been rehoused under *renal tubular secretion* (GO:0097254) (or one of its children) or removed if the paper supporting the annotation did not allow one to infer the process that affected the circulating levels of urea or creatinine.
- **Reaction mechanisms:**  *primary charge separation* (GO:0009766) and *enzyme active site formation* (GO:0018307) were obsoleted because they represent substeps of reactions which are beyond the scope of GO.
- **Protein-modifying activity terms that mention specific substrates**, for example *[cytochrome c]-arginine N-methyltransferase activity* (GO:0016275), which is captured by the more general *arginine N-methyltransferase activity* (GO:0016274). Substrates can be captured with the “has input” relationship in GO-CAM models and in annotation extensions. The exception to this is the histone code: for GO to represent this important mechanism of gene expression and chromatin structure mechanism, specific activities are created for known histone modifications, for example *histone H2AR3 methyltransferase activity* (GO:0070612) and *histone H3T3 kinase activity* (GO:0072354).
- **Experimental assays and nonphysiological substrates**: some experiments are easier to perform using analogs of physiological substrates. Because GO terms should represent *in vivo* functions, we have removed some terms that represent an experiment rather than its biological conclusion. An example is *rubidium ion transport* (GO:0035826): rubidium is used as a tracer for potassium ions (Gill *et al.* 2004) but has no physiological role in itself. Another example is *regulation of nucleosome density* (GO:0060303), which measures the degree of compaction of chromatin, and is a readout for heterochromatin assembly or disassembly.

Concomitantly with these term obsoletions, many new terms have been added to the ontology in the past two years. An example is *molecular condensate scaffold activity* (GO:0140693) for proteins that nucleate condensates that mediate liquid phase transition. This latter term represents a recent advance in the understanding of the organization of cellular biochemistry (Banani *et al.* 2017).

We have also clarified the level of specificity at which MF terms should be represented in GO. For example, we now strive to create GO terms that represent the range of *in vivo* substrate specificity of an enzyme or transporter. This is in contrast to earlier guidelines, in which a GO term was created for each separate molecular substrate tested in a single, isolated experimental assay or result, which could include nonphysiological substrates. With recent improvements in experimental technologies and practices, it is now often possible to annotate with a concept that more closely matches the biological substrate specificity range of a protein. Therefore, while GO makes cross-references to Enzyme Commission (EC) (McDonald and Tipton 2014), Rhea (Bansal *et al.* 2022), Kyoto Encyclopedia of Genes and Genomes (KEGG) (Kanehisa *et al.* 2022), and MetaCyc (Altman *et al.* 2013), GO does not necessarily create a different term for each of the reactions represented in these resources for each substrate on which a MF acts. For example, the GO term *3-oxoacyl-[acyl-carrier-protein] reductase (NADPH) activity* (GO:0004316) represents the fact that the same gene product has a broad specificity toward 3-oxo-acyl groups, and therefore, we have obsoleted the more specific GO terms that refer to only one specific substrate, such as *3-oxo-cis-Delta9-hexadecenoyl-[acp] reductase activity* (GO:0102072), *3-oxo-glutaryl-[acp] methyl ester reductase activity* (GO:0102131), and *3-oxo-pimeloyl-[acp] methyl ester reductase activity* (GO:0102132). For broad specificity enzymes and transporters, the activity on a specific substrate in a specific pathway can be captured by biocurators in a GO-CAM (Thomas *et al.* 2019) or an annotation extension (Gene Ontology Consortium 2010) rather than in a GO term.

### Annotations

A GO annotation is a statement asserting that a particular gene or gene product has a particular functional characteristic (GO term); examples are shown in Fig. 1b. New annotations are continually added to the knowledgebase. In the past two years, experimentally supported gene function annotations have been added from over 10,000 scientific papers. As of November 2022, the GO knowledgebase contains experimental knowledge from almost 173,000 papers. GO annotations derived from experimental data are added primarily by the annotation groups in the GO Consortium, which typically curate biological knowledge by organism (Table 2).

**Table 2.** Groups contributing literature-based annotations. Includes all annotations traceable to the literature (EXP, including HTP, TAS, NAS, and IC; see http://geneontology.org/docs/guide-go-evidence-codes; see below for information). Direct annotations to the term “protein binding” are listed separately, since without information about interacting partner(s), protein binding represents an activity that most proteins possess, and therefore the GO class itself provides little information (see text for further description). The statistics for groups that have contributed more than 700 manual annotations. Other contributing groups include the following: HGNC, JaponicusDB, PHI-base, PAMGO, JCVI, MENGO, and GDB. Current GO Consortium members are labeled with an asterisk. See http://geneontology.org/docs/annotation-contributors/ for more details.

Group	Organism or area of focus	Number of literature-based annotations, excluding direct protein binding	Number of literature-based annotations directly to protein binding
UniProt* (UniProt: the universal protein knowledgebase 2017)	Human and also a wide variety of organisms not covered by other GOC members	185,121	30,927
MGI* (Bult *et al*. 2019)	Mouse	106,435	8,051
Reactome* (Fabregat *et al*. 2018)	Human pathways	92,178	6
TAIR* (Lamesch *et al*. 2012)	*A. thaliana* (model plant)	64,633	4,695
FlyBase* (Sian *et al*. 2022)	*D. melanogaster* (fruit fly)	55,203	892
UCL*	Human	54,595	2,935
RGD* (Smith *et al*. 2020)	Rat	47,694	1,894
SGD* (Lang *et al*. 2018)	*Saccharomyces cerevisiae* (Baker's yeast)	48,811	165
ZFIN* (Howe *et al*. 2021)	Zebrafish	28,261	488
PomBase* (Harris et al. 2022)	*Schizosaccharomyces pombe* (fission yeast)	26,128	2,201
GeneDB	Microbial pathogens	23,884	756
ComplexPortal* (Meldal *et al*. 2019)	Protein complexes	18,343	0
WormBase* (Davis *et al*. 2022)	*C. elegans* (nematode)	17,171	560
CGD* (Skrzypek *et al*. 2017)	*Candida albicans* (yeast pathogen)	17,113	0
EcoCyc* (Keseler *et al*. 2017)	*E. coli* (bacterium)	13,372	829
AgBase	Agricultural animals, primarily chicken	11,198	1,110
dictyBase* (Basu *et al*. 2015)	*Dictyostelium discoideum* (slime mold)	9,615	844
HPA	Human protein subcellular localization	9,963	0
SynGO (Koopmans *et al*. 2019)	Neuron–neuron synapses	9,552	0
PINC	Human and mouse	6,746	0
MTBBASE	*Mycobacterium tuberculosis* (bacterial pathogen)	6,160	463
IntAct* (Del Toro et al. 2022)	Protein–protein interactions	4,849	216,488
CAFA (Radivojac *et al*. 2013)	Various	4,818	371
CACAO* (Ramsey *et al*. 2021)	Various	4,382	0
AspGD (Cerqueira *et al*. 2014)	*Aspergillus niger* (fungal pathogen)	4,099	0
PseudoCAP (Winsor *et al*. 2005)	*Pseudomonas aeruginosa* (bacterium)	2,323	0
EcoliWiki* (McIntosh *et al*. 2012)	*E. coli* (bacterium)	2,123	55
TIGR	Bacteria	2,150	0
GO_Central*	Various	3,643	160
CollecTF	Bacterial transcription factors	1,850	0
NTNU_SB	Human, mouse, and rat transcription factors	1,733	0
GR	Rice	1,260	0
SGN	Tomato	1,255	0
DisProt	Disordered proteins	933	156
Xenbase* (Fortriede *et al*. 2020)	Xenopus (frog)	731	0

GO annotations are also regularly reviewed and may be edited or removed from the knowledgebase for various reasons, particularly when ontology terms are revised (see “Ontology” section above) or when annotations are invalidated by later experimental data. Annotations to terms that will be obsoleted are manually reviewed and annotations are made to a different term whenever possible. For example, when we edited the ontology for histone modifications, over 2,000 annotations to the obsoleted terms were manually reviewed, and histone modifying enzymes were reannotated to the appropriate MF term, while annotations from indirect effects were either removed or reannotated to different, appropriate GO terms. More minor annotation reviews occur regularly.

The Phylogenetic Annotation with the GO project (see below) involves an integrated biocurator review of annotations that has provided additional quality control. The GO user community also plays an important role in identifying incorrect annotations. Because each annotation can be traced to the published paper containing the underlying evidence or describing a method used to infer the annotation, users can quickly verify the accuracy of a given annotation. Potential errors can be reported by clicking on the “Help” link at the top of the GO homepage (http://geneontology.org). In addition, authors of a paper used to create GO annotations can easily retrieve and review all annotations from a given paper and suggest changes; this can be done from the PubMed abstract page (e.g. the PubMed page https://pubmed.ncbi.nlm.nih.gov/20516198/ (Lydeard *et al.* 2010)) by clicking on LinkOut and then the “Gene Ontology” link.

### Phylogenetic annotations as a source of highly reviewed annotations

The Phylogenetic Annotation using GO (PAN-GO) project creates a set of biocurator-reviewed, selected GO annotations. The PAN-GO process is described in detail in Gaudet *et al.* 2011. Briefly, using the PAINT software tool, a biocurator reviews all experimentally supported GO annotations collected for all members of a protein family, in the context of a phylogenetic tree from the PANTHER resource (Thomas *et al.* 2022). They then select the most informative and nonredundant GO terms that represent the gene's functional characteristics. Biocurators then model the evolution of these characteristics in the tree by specifying branches along which the GO terms were gained or lost, taking into account events such as duplications, mutations, horizontal gene transfers, and taxonomic specificity. This allows for different members of the same family to be annotated with different GO terms when justified by the experimental data. All PAN-GO annotations can be traced to experimental evidence in one or more related genes. To date, a total of 8,196 protein families (out of 11,719 families with experimental data) have been curated. The PAN-GO curation effort has prioritized human gene-containing families, though many other families have also been curated. As a result, annotation coverage of a genome generally depends on how closely related it is to humans. PAN-GO annotations are available for 82% of human genes (compared with 68% with experimental evidence alone). Other vertebrate genomes have similarly high coverage, with genomes from other taxa covered at lower but still substantial levels (Tables 3 and 4). PAN-GO annotations are updated at each GO release and are included in the standard, downloadable GO annotation files. These annotations can be identified by the “IBA” (inferred from biological ancestor) evidence code and are available for the 142 organisms included in PANTHER gene families (http://pantherdb.org/panther/summaryStats.jsp).

**Table 3.** Genome coverage of PAN-GO annotations. Percentage of protein-coding genes with at least one PAN-GO-reviewed annotation, for different taxonomic groups.

Taxonomic group	Number of individually reviewed, annotated genomes	Gene coverage of annotations
Vertebrates	19	66%–83%
Invertebrates	15	40%–68%
Fungi	14	32%–76%
Plants	40	28%–51%
Protists, alveolates, and amoebae	11	19%–46%
Archaea	8	23%–34%
Bacteria	35	20%–57%

**Table 4.** Number of PAN-GO annotations for selected genomes.

Genome	Total IBA annotations	MF annotations	BP annotations	CC annotations
*Danio rerio*	82,855	23,049	33,888	25,918
*Mus musculus*	72,554	19,832	29,947	22,775
*Rattus norvegicus*	71,276	19,792	28,738	22,746
*Homo sapiens*	68,695	18,537	28,177	21,981
*Gallus gallus*	59,293	15,847	24,010	19,436
*Xenopus tropicalis*	43,232	12,687	16,735	13,810
*Arabidopsis thaliana*	37,509	12,106	12,922	12,481
*Caenorhabditis elegans*	31,093	9,021	11,728	10,344
*Drosophila melanogaster*	30,331	8,628	11,202	10,501
*Dictyostelium discoideum*	18,966	5,556	6,815	6,595
*Saccharomyces cerevisiae*	15,675	4,286	5,763	5,626
*Schizosaccharomyces pombe*	13,723	3,719	4,953	5,051
*Escherichia coli*	5,681	2,171	1,916	1,594

### Protein binding and protein-containing complex annotations

We suggest that users should be particularly cautious when using GO annotations directly to the term *protein binding* (GO:0005515; see Table 2). These are highly specific annotations that include the protein binding partner in another field of the annotation (not in the GO term itself) and should not be used in applications such as gene set enrichment analysis. Instead, they are recommended for applications such as protein–protein interaction network construction for human proteins (which represent the vast majority of direct protein binding annotations in the knowledgebase). Since all protein functions encompass some type of binding (to a substrate or to another protein), GO strives to describe the molecular activity of proteins using at least one term that is not only under the *binding* branch of GO; see also the “noncatalytic MF” section above. Therefore, *binding* (GO:0005488) in isolation can be considered a limited functional description and is represented as a distinct branch of GO MF.

### Annotation evidence

All annotations are supported by evidence, comprising two fields in the annotation file (Fig. 1b): an *evidence code* that describes the type of evidence, and a *reference* that lists a persistent identifier for tracing the source (provenance) of the original data. It has often been asserted that the most reliable annotations are those made using an experimental evidence code. However, we suggest that users take into account the type of experimental evidence and the level of review of the annotation (Table 5). Some types of experimental evidence, such as inference from a gene expression pattern (IEP), mutant phenotype (IMP), or genetic interaction (IGI), can often be suggestive of function but not definitive when considered in isolation; other annotations for the same gene are often useful to help interpret these annotations. “High-throughput” evidence codes should be treated with particular care. These codes (beginning with the letter H) denote experiments in which many genes are analyzed at the same time, and these annotations are not individually reviewed by either the paper's authors or GO Consortium biocurator (Attrill *et al.* 2019). Conversely, many nonexperimental evidence types are carefully reviewed by experts. Phylogenetic annotations (IBA evidence code) are based on integration and expert assessment of experimental annotations and thus are *individually reviewed* twice: once in making the annotation from published experimental results and once in the context of all annotations for related genes (Gaudet *et al.* 2011). While annotations using the Inferred from Electronic Annotation (IEA) evidence code are considered automated, most implement expert review of a subset of annotations to minimize false positives (for example, UniRule (MacDougall *et al.* 2021) and InterPro2GO (Paysan-Lafosse *et al.* 2022)). The GOC considers these annotations to be accurate though they are often less specific than other annotations.

**Table 5.** GO evidence codes + reference combinations. Users should consider both the type of evidence and the review level. GO internal references (starting with GO_REF:) describe specific annotation methods and are available at https://github.com/geneontology/go-site/tree/master/metadata/gorefs/README.md.

Evidence code	Reference	Evidence-type description	Review-level description
Inferred from direct assay (IDA)	Scientific publication providing experimental data	Experimental, most direct evidence for function	Individually expert-reviewed
Inferred from mutant phenotype (IMP)	Scientific publication providing experimental data	Experimental, from a perturbation in the normal function	Individually expert-reviewed
Inferred from genetic interaction (IGI)	Scientific publication providing experimental data	Experimental, from perturbations in normal functions of more than one gene	Individually expert-reviewed
Inferred from expression pattern (IEP)	Scientific publication providing experimental data	Experimental, used only for biological process annotations, from comparison with genes of known function	Individually expert-reviewed
Inferred from protein interaction (IPI)	Scientific publication providing experimental data	Experimental, used only for annotations to protein binding terms	Individually expert-reviewed
Inferred from high throughput direct assay (HDA)	Scientific publication providing experimental data	Experimental (high throughput), direct	Not individually reviewed; expert review methodology to exclude high false positive rate observations
Inferred from high throughput mutant phenotype (HMP)	Scientific publication providing experimental data	Experimental (high throughput), mutant phenotype	Not individually reviewed; expert review to exclude high false positive rate observations
Inferred from high throughput genetic interaction (HGI)	Scientific publication providing experimental data	Experimental (high throughput), genetic interaction	Not individually reviewed; expert review to exclude high false positive rate observations
Inferred from high throughput expression pattern (HEP)	Scientific publication providing experimental data	Experimental (high throughput), expression pattern	Not individually reviewed; expert review to exclude high false positive rate observations
Inferred from biological ancestor (IBA)	(Gaudet *et al*. 2011)	Homology, from experimental evidence propagated through a phylogenetic tree and/or from direct experimental evidence	Individually expert-reviewed in the context of all experimental annotations for related genes
Inferred from sequence similarity (ISS)	Scientific publication providing sequence similarity evidence	Homology, from experimental evidence propagated from one gene to one related gene, asserted in the publication	Individually expert-reviewed in the context of all experimental annotations for related genes
Inferred from sequence similarity (ISS)	GO_REF:0000024	Homology, from experimental evidence propagated from one gene to one related gene, asserted by a biocurator	Individually expert-reviewed
Inferred from sequence orthology (ISO)	Scientific publication providing orthology evidence	Homology, from experimental evidence propagated from one gene to one orthologous gene	Individually expert-reviewed
Inferred from sequence Orthology	GO_REF:0000008	Homology, from experimental evidence propagated from one mammalian gene to one orthologous mouse gene	Individually expert-reviewed
ISO, Inferred from Sequence orthology (ISO)	GO_REF:0000024	Homology, from experimental evidence propagated from one gene to one orthologous gene	Individually expert-reviewed
Inferred from sequence orthology (ISO)	GO_REF:0000096	Homology, from experimental evidence propagated from one gene to one orthologous gene among human, mouse, and rat orthologs	Not individually reviewed; orthology manually reviewed
Inferred from sequence orthology (ISO)	GO_REF:0000101	Homology, from experimental evidence propagated from one gene to one orthologous gene	Not individually reviewed; orthology computed using OrthoMCL
Inferred from electronic annotation (IEA)	GO_REF:0000107	Homology, from experimental evidence propagated from one gene to one orthologous gene	Not individually reviewed; one-to-one orthology computed using Ensembl Compara phylogenetic trees
Inferred from electronic annotation (IEA)	GO_REF:0000002	Homology, from a hit to an InterPro signature	Not individually reviewed; expert review of annotations of signatures to ensure low or no false positives
Inferred from electronic annotation (IEA)	GO_REF:0000003	Imported from another resource, from mapping an EC number assigned in UniProt	Not individually reviewed; expert review of mappings; EC assignments are manually reviewed for Swiss-Prot and computationally inferred for TrEMBL
Inferred from electronic annotation (IEA)	GO_REF:0000004	Imported from another resource, from mapping a manually assigned Swiss-Prot keyword	Not individually reviewed; expert review of keywords and mappings
Inferred from electronic annotation (IEA)	GO_REF:0000104	Homology, from manually curated UniRule	Not individually reviewed; expert curation of UniRules to ensure low or no false positives
Inferred from electronic annotation (IEA)	GO_REF:0000108	Logical assertion using the ontology, from asserted relation between different aspects of GO	Not individually reviewed; expert curation of ontology links
Inferred from electronic annotation (IEA)	GO_REF:0000117	Computational, from machine learning	Not individually reviewed; assigned by machine learning from curated training sets
Traceable author statement (TAS)	Scientific publication citing original data	From a published statement referencing experimental evidence in a different paper	Individually reviewed
Nontraceable author statement (NAS)	Scientific publication with general biological knowledge statement	From an unreferenced published statement	Individually reviewed

GO evidence codes correspond to a subset of the terms found in the Evidence and Conclusion Ontology (ECO) (Nadendla *et al.* 2022). Combinations of particular GO internal references (GO_REFs) and evidence codes are also mapped to specific ECO terms (https://github.com/evidenceontology/evidenceontology/blob/master/gaf-eco-mapping.txt). Users needing to map granular ECO terms to GO evidence code abbreviations can use the mapping file provided by ECO (https://github.com/evidenceontology/evidenceontology/blob/master/gaf-eco-mapping-derived.txt).

### GO causal activity models

GO-CAMs are models of causal influences between gene products (Thomas *et al.* 2019) or pathways. More precisely, a GO-CAM links the activities (GO MFs) of gene products together by causal relations that specify the effect of one activity on the other. Each element of a GO-CAM is an instance of an ontology class or other standard database identifiers, so GO-CAMs are highly structured and amenable to computational analysis. The basic unit of a GO-CAM is a “gene product activity unit,” which combines a GO MF annotation (molecular activity), together with GO CC (location) and GO BP (larger functional module) annotations that provide the biological context of the activity. The context can be further specified with other ontologies to capture the cell type [using the Cell Type Ontology (Diehl *et al.* 2016)], tissue/anatomical location (using several different ontologies depending on the species, e.g. Uberon (Mungall *et al.* 2012) for most vertebrates, other metazoan ontologies such as the *Drosophila* anatomy ontology (Costa *et al.* 2013), *Caenorhabditis elegans* anatomy ontology (Lee and Sternberg 2003), or nonanimal ontologies as the Plant Ontology (Cooper and Jaiswal 2016), or a temporal period (e.g. GO biological phase). Activity units are linked together by causal relationships from the Relations Ontology (Smith *et al.* 2005) to capture how they interact to impact larger pathways, modules, or processes.

**Fig. 2.**
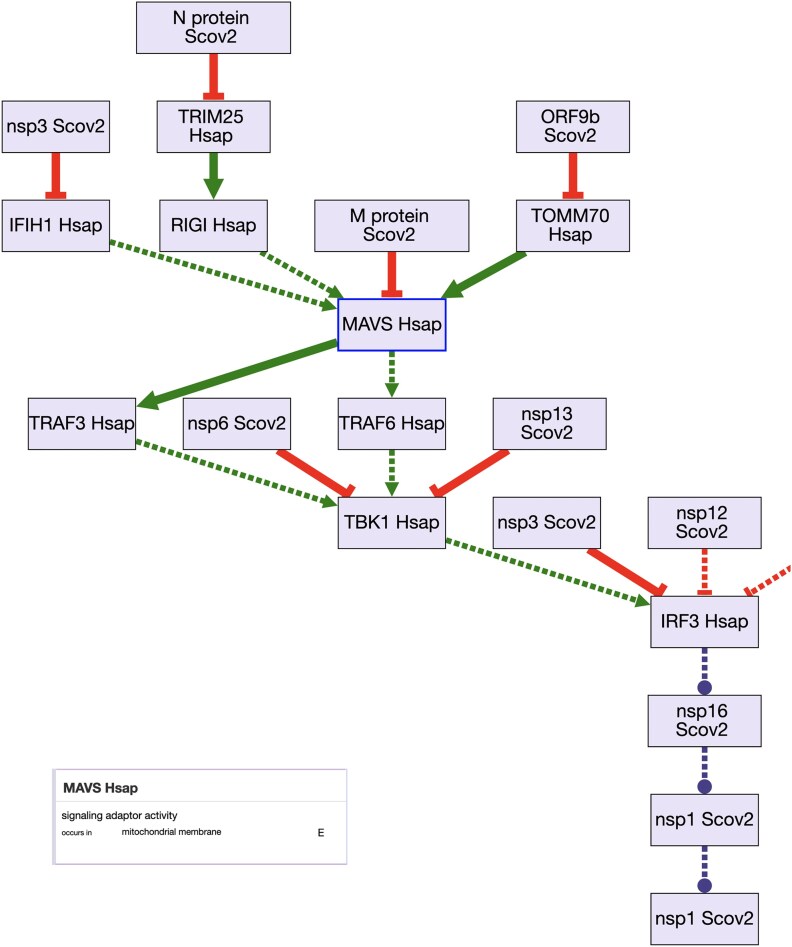
GO-CAM model of the SARS-CoV2—host interactions as displayed using the GO-CAM Pathway Widget (code available at https://github.com/geneontology/wc-gocam-viz) on the Alliance of Genome Resources gene pages (https://www.alliancegenome.org/gene/HGNC:20144#pathways). The model includes proteins from both humans (Hsap) and the SARS-CoV-2 virus (Scov2). A simplified representation of the causal model is shown on the main figure, which is simplified by labeling each activity with the gene and organism. The model includes many additional details, which are displayed as “cards;” the information for MAVS activity (inset) which normally acts as a signaling adaptor located in the mitochondrial membrane. MAVS activity is suppressed directly by the SARS-CoV-2M protein and indirectly by other SARS-CoV-2 proteins. Each of the “E” symbols on the right-hand side can be clicked to see the evidence for each assertion in the model.

As of November 2022, GO Consortium annotation groups have created over 300 GO-CAM models that describe molecular pathways (defined as containing at least three distinct gene product activities linked into a causal chain). These models reflect curation priorities of the contributing groups. Most of the available GO-CAMs are for processes in human or mouse, with a limited number in zebrafish, *Drosophila melanogaster*, and *C. elegans*. Many of the human GO-CAMs describe chromatin-mediated regulation of gene expression and immune response pathways, while the mouse GO-CAMs focus on metabolic and signaling pathways. GO-CAMs are accessible from the GO website homepage, by clicking on the “Browse GO-CAMs” link. GO-CAMs can be viewed as pathway diagrams (Fig. 2) and are currently available on GitHub at https://github.com/geneontology/noctua-models.

## Community collaborations

The GO Consortium collaborates with experts in specific areas of molecular and cellular biology to systematically update and improve their representation in the ontology and the corresponding GO annotations and GO-CAMs. We recently revised the representation for transcription factors and transcriptional regulation in collaboration with the GREEKC Consortium (Kuiper *et al.* 2022). Additional collaborative projects include working with the DisProt project (Quaglia *et al.* 2022) on improving the ontology and annotations for intrinsically disordered proteins (IDPs); revising processes that involve molecular pathways between interacting species, such as viral infection processes; and integrating the GO and annotations with external biochemical databases.

In 2021, the GO started a collaboration with DisProt (https://disprot.org/)—the gold standard database of manually curated annotations from the literature for IDPs. IDPs lack a stable three-dimensional structure and are characterized by highly flexible and unstructured segments, i.e. intrinsically disordered regions (IDRs). DisProt has developed a custom ontology, the Intrinsically Disordered Proteins Ontology (IDPO), and used it to annotate the structural states of IDPs. The GO Consortium and DisProt have collaborated to refactor IDPO and map the IDPO terms to GO terms whenever possible (those related to functions and interactions of IDPs). The collaboration between the GO Consortium and the DisProt database included the creation and addition of new GO terms to align with already existing IDPO terms that were not yet available in GO. These newly created terms also include the *MF activator* (GO:0140677) and *MF inhibitor* (GO:0140678) terms, used to annotate MF regulators that activate/inhibit or increase/decrease the activities of their targets via noncovalent binding that does not result in covalent modification to the target. This collaboration resulted in more accurate and detailed annotation of the modes of action of IDPs, e.g. *localization* (GO:0051179, IDPO:00010) and *DNA binding* (GO:0003677, IDPO:00065), as well as providing GO annotations. Currently, more than 1,000 expert-curated annotations from DisProt are available in the GO knowledgebase, comprising more than 860 MFs, 200 BPs, and 10 CC annotations. The only terms in IDPO that could not be mapped to GO were those describing self-regulatory (e.g. *self-activation* and *self-inhibition*) and intrinsic disorder-specific functions (i.e. *entropic chains*), so these annotations are available only in DisProt.

### Multiorganism interactions

A group that includes experts from within and outside the GO Consortium has been working together to improve and simplify the representation of interactions between organisms, including medically and agriculturally important host–pathogen interactions. Examples of these interactions include how a symbiont such as a virus enters its host, how the host's immune response recognizes and defends the body against a potentially harmful organism, and also beneficial interactions such as how plants form a symbiosis with nitrogen-fixing bacteria. The goal of this project is to revise the host–symbiont branch of GO *BP* to reflect the current scientific knowledge in the field and to ensure that genes are properly annotated to the new ontology terms and structure, building on previous work undertaken as part of the PAMGO consortium (Tyler *et al.* 2009). Symbionts in GO are broadly defined to include pathogens that infect a host organism. We expect that this revision will improve GO-based analyses of molecular studies of pathogens, the mechanisms by which they infect host cells, and host response processes. A major change is that the branch of GO under *BP involved in interspecies interaction between organisms* (GO:0044419) has been reorganized. It now reflects important concepts such as the types of biological programs used by symbionts to enable infection and by hosts to prevent or manage infection, such as *disruption of CC of another organism* (GO:0140975), *formation of structure involved in a symbiotic process* (GO:0044111), *killing of cells of another organism* (GO:0031640), and *modulation of process of another organism* (GO:0035821). Each of these terms has multiple, more specific subclass terms.

One challenge in this area was that some previous GO annotations for pathogen genes used terms that apply to normal host processes, such as regulation of defense response processes. Thus, it was not clear whether the pathogen gene was regulating its own defense process or that of a host. With the new ontology terms and structure, these distinctions are clear for both GO biocurators and users of GO. In general, it was important to clearly represent that certain symbiont-initiated processes hijack various host cellular processes. This includes mechanisms to enter and exit the cell, either by binding to host membrane proteins or using the intracellular transport machinery and using the host cellular machinery for genome replication, as well as transcription and translation. We have obsoleted terms that do not clearly distinguish hijacking with the functions that a host gene performs for the host organism, such as *dissemination or transmission of symbiont from host by vector* (GO:0044008) and *positive regulation of viral release from host cell* (GO:1902188). Conversely, a pathogenic symbiont triggers innate responses in the host that are not the evolved role of these symbiont proteins, such as *induction by symbiont of host cytokine production* (GO:0036523) and *pathogen-associated molecular pattern-dependent induction by symbiont of host innate immune response* (GO:0052033)—these are not functions that a symbiont protein performs to enable its own survival and reproduction.

### Integration with biochemical knowledgebases

For accurate representation of biochemical aspects of gene function, we work closely with the Rhea database of reactions (Bansal *et al.* 2022) and the ChEBI ontology of chemical entities (Hastings *et al.* 2016). Rhea provides precise representations of *in vivo* biochemical reactions, including precise chemical entity participants and their stoichiometry. Rhea uses ChEBI terms to represent chemical entities in a standardized, consistent manner. The Rhea database overlaps in content with the catalytic activity branch of the GO but provides additional detailed reaction information and in some cases provides additional specificity. We have improved GO mappings to Rhea, which now covers 4,399 GO catalytic activities (in the MF branch of GO). These mappings allow for nonexact matches when the chemical specificity differs between GO and Rhea. For example, Rhea has two reactions, each referring to a different type of beta glucoside (RHEA:69647 and RHEA:69655, narrow match), whereas GO:0008422, beta-glucosidase activity, covers both substrates, as no known enzyme is specific for just one of them. We have recently used the Rhea-GO mappings to include additional linkages between GO MF terms and ChEBI terms in the go-plus release (see below). Previously, ChEBI terms were linked only to general terms in the GO BP branch (e.g. between folate transport and folate), but the additional Rhea linkages have added a total of 4,334 distinct chemical entities linked via 20,307 relationships. The extensive linkage to chemical entities opens opportunities for using GO in other applications, e.g. metabolomics analyses.

## Accessing and downloading GO data

### Browsing GO and its annotations

GO and associated annotations can be searched directly from the GO home page (http://geneontology.org/), queried using the AmiGO browser (http://amigo.geneontology.org/amigo) or the QuickGO tool (https://www.ebi.ac.uk/QuickGO/) (Munoz-Torres and Carbon 2017). Gene set enrichment analysis is also directly accessible from the GO home page, which launches the PANTHER gene analysis tool at http://pantherdb.org/webservices/go/overrep.jsp (Mi *et al.* 2019).

### Ontology downloads

GO provides three editions of the ontology on the download page (http://geneontology.org/docs/download-ontology/) to accommodate various applications: go-basic, go, and go-plus (Table 6). All GO terms, including obsolete terms and term metadata such as definitions, cross-references, and synonyms, are available in all three editions. These editions differ in the set of relations they contain:

- **go-basic** contains the types of information that has been available for GO from the beginning of the project; hence, it only contains is a, part of, regulates, negatively regulates, and positively regulates relationships and excludes relationships that cross different aspects (BP, MF, or CC) of the ontology. This edition of the ontology is guaranteed to be acyclic and can safely be used to selectively propagate annotations across any relation. It is recommended for most GO-based software tools.
- **go** additionally includes has part and occurs in relationships that link terms across different aspects of the ontology (for example, a BP can have a *has part* relation to a MF term or an *occurs in* relation to a CC). This edition is not acyclic and annotations should not be propagated across all the relationship types that it contains. This edition should not be used in most software tools that rely on the GO.
- **go-plus** is the fully axiomatized edition of the ontology and includes cross-ontology relationships to external ontologies including ChEBI, Cell Ontology, and Uberon.

**Table 6.** GO editions. Editions are distinguished by the relations and metadata that they include. All editions are updated at each GO release. External ontologies used in GO include the following: ChEBI, Uberon (Haendel *et al.* 2014), Relation Ontology (Smith *et al.* 2005), Cell Ontology (Diehl *et al.* 2016), Sequence Ontology (Mungall, Batchelor and Eilbeck 2011), Dicty Anatomy, CARO (Haendel *et al.* 2008), Fungal Anatomy Ontology (Fungal-Anatomy-Ontology, 2020), Plant Ontology (Walls *et al.* 2019), PATO (Gkoutos, Schofield and Hoehndorf 2018), and Protein Ontology (Natale *et al.* 2017).

GO edition	Format(s)	Relations included	Links to other ontologies
go-basic	OBO	Is a, part of, regulates, negatively regulates, and positively regulates	Not available
go	OBO and OWL-RDF/XML	Same as go-basic, plus has part, and occurs in	Not available
go-plus	OWL-RDF/XML		ChEBI, Uberon, Cell Ontology, Sequence Ontology, Dicty Anatomy, CARO, Fungal Anatomy Ontology, Plant Ontology, PATO, and Protein Ontology

### Ontology subsets (GO slims)

GO subsets are condensed versions of the GO containing a portion of the terms, which are specified by tags within the ontology files that indicate if a given term is a member of a particular subset. GO subsets are particularly useful for providing a global overview of the functions of all the genes in a genome, and even for all the functions of a single gene. GO subsets are particularly useful for providing a global overview of the range of functions and processes found in a given clade or organism's genome. We have recently revised the “GO Generic subset,” a subset maintained by the GO Consortium that aims to be general and applicable to any species. We have tested that the subset covers as many gene products as possible in various organisms (human, *D. melanogaster*, fission yeast, *Arabidopsis thaliana*, and *Escherichia coli*) with as little redundancy as possible. This new GO Generic Subset contains 75 BP terms, 40 MF terms, and 29 CC terms. The GO generic subset can be accessed at http://current.geneontology.org/ontology/subsets/goslim_generic.obo. Versions in .owl, .json, and .tsv are also available from http://current.geneontology.org/ontology/subsets/index.html.

**Fig. 3.**
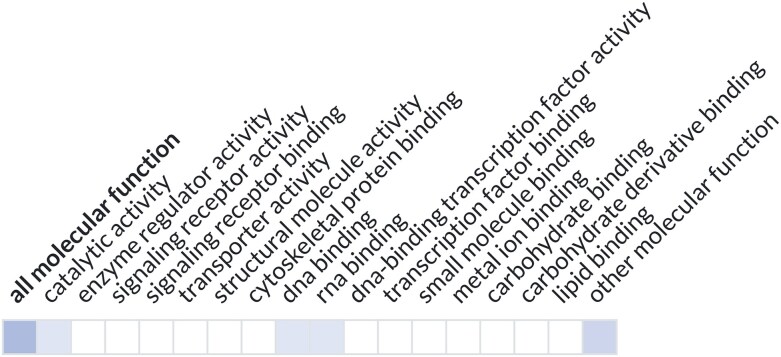
Alliance ribbon view for the yeast RPB7 gene. High-level GO categories annotated are shown in shaded squares (https://www.alliancegenome.org/gene/SGD:S000002812); darker shading indicates more annotations in that category.

As part of the Alliance of Genome Resources, we have developed a widget that provides a graphical visualization of a gene's function in a “ribbon”-like display (Fig. 3). The widget can be customized to use any GO subset and uses the goslim_agr subset by default. This widget is implemented in the Alliance gene pages and in the UniProt entry pages. It accesses GO annotations using the GO API (application programming interface) and can be easily added to any webpage.

### GO annotations

The two major sites for downloading GO annotations are geneontology.org and UniProt-GOA. geneontology.org is the website developed by the GO Consortium. This downloads site (http://current.geneontology.org/products/pages/downloads.html) provides a total of 7.5 million human and model organism annotations contributed by multiple groups. It contains all manually reviewed GO annotations and electronic (computationally predicted) annotations for the most commonly used organisms. For model organisms, all annotations use gene identifiers from the authoritative database (for example, FlyBase (FBgn), WormBase (WBGene), and SGD (S)). Human and other organisms without an authoritative dedicated database are represented by UniProtKB accession numbers. For these organisms, the GO website provides annotations to UniProt reference proteomes (https://www.uniprot.org/help/reference_proteome), which are generally one entry per gene, thus limiting redundancy in annotations. UniProt-GOA (https://www.ebi.ac.uk/GOA/uniprot_release) contains 1 billion annotations for all entries in UniProt (1,264,340 taxa), covering both reviewed (Swiss-Prot) entries of UniProt, and unreviewed (TrEMBL) entries. All annotations for the model organism genes are converted to UniProt protein identifiers. For most organisms, all annotations are electronic annotations generated via various pipelines (see above for evidence codes and references for different methods). In addition to these resources, GO annotations are also viewable in a number of biological databases, including model organism databases, UniProt (UniProt: the universal protein knowledgebase 2017), NCBI (Sayers *et al.* 2020), and The Alliance of Genome Resources (Alliance of Genome Resources Consortium 2022). These sites show GO annotations in the broader context of a gene product's expression pattern, phenotypes, metabolic and signaling pathways, etc.

### Conclusions and future directions

The extensive and wide-ranging use of the GO knowledgebase, evidenced by its recent, peer-reviewed designation as a Global Core Biodata Resource (https://globalbiodata.org/scientific-activities/global-core-biodata-resources/), demands its continued development and expansion. We are focusing on several high-priority areas of development for the near future. For pathways, we will continue to accumulate GO-CAM models. The UniProt/Swiss-Prot curation team has ramped up their production of GO-CAM models and we expect to add models at a rapid rate. In parallel, we have started converting Reactome pathways into GO-CAMs (Good *et al.* 2021) and expect to release GO-CAM representations of most Reactome metabolic pathways in the near future. This will provide a complementary, causal flow representation of the chemical reaction-centered representation in Reactome. Conversion of Reactome signaling pathways is more challenging and will be released somewhat later. We are also working on converting the YeastPathways resource (https://pathways.yeastgenome.org) into GO-CAMs, making a large number of yeast metabolic pathways available. The increasing number of GO-CAM models will allow us to expand on the utility of these highly structured pathway and process representations. Some potential areas are automated pathway visualization, using the causal links and more granular gene sets to enhance enrichment analysis, and better generation of automated descriptions of gene function (e.g. Kishore *et al.* 2020).

With respect to ontology development, in addition to continuing to revise the ontology in response to recent discoveries, we see an immediate need for clearly delineating the level of biological organization at which a function is described. This includes distinguishing MFs from BPs, and distinguishing BPs that occur at the level of individual cells, versus those that occur at the level of multicellular organisms. For example, the term "homeostasis"—the maintenance of a roughly steady level of a molecule or ion—is used very broadly in the literature to refer to both processes that maintain a steady-state level within a cell and processes that maintain a steady state in blood or other fluid that is transported within a multicellular organism. Even in some publications, it is difficult to know which type of homeostasis is being tested.

We will continue to make the GO knowledgebase easier to use and more community-driven. One near-term priority is to make annotations available for download by species, with a single identifier for each distinct gene. We are also planning to create quick-start guides for common GO use cases, in both written and video forms. The immense user base of the GO and the need for much improvement and extension drives us to consider how to expand the number of people that contribute to the GO. From its inception, the GO has been a large, open, community project. However, we are planning additional routes through which the broader GO user community can contribute their expert feedback and knowledge to GO, improving the resource for all users. For now, users are encouraged to contact the GO Helpdesk (http://help.geneontology.org/) with any questions or to report any GO ontology terms or annotations that may be inaccurate or difficult to interpret.

## Data Availability

All GO code and resources are freely available for download and reuse. Software (https://github.com/geneontology/) is under the BSD 3-Clause open-source license. Downloads are available under the CC BY 4.0 license from http://geneontology.org/docs/downloads/

## Summary

The Gene Ontology (GO) knowledgebase has broad applications in genetic and genomic research and has been continually updated and improved for more than 20 years. We describe the latest improvements to the GO resource and give an overview of the current contents of the knowledgebase. We also include guidance on the use of GO, annotations, and the growing number of pathways represented as GO-Causal Activity Models (GO-CAMs).

## References

[iyad031-B1] Alliance of Genome Resources Consortium . Harmonizing model organism data in the Alliance of Genome Resources. Genetics. 2022;220:iyac022. doi:10.1093/genetics/iyac022. 35380658 PMC8982023

[iyad031-B2] Altman T, Travers M, Kothari A, Caspi R, Karp PD. A systematic comparison of the MetaCyc and KEGG pathway databases. BMC Bioinformatics. 2013;14(1):112. doi:10.1186/1471-2105-14-112. 23530693 PMC3665663

[iyad031-B3] Ambrus JL Jr, Pippin J, Joseph A, Xu C, Blumenthal D, Tamayo A, Claypool K, McCourt D, Srikiatchatochorn A, Ford RJ. Identification of a cDNA for a human high-molecular-weight B-cell growth factor. Proc Natl Acad Sci U S A. 1993;90(13):6330–6334. doi:10.1073/pnas.90.13.6330. 8327514 PMC46922

[iyad031-B4] Ambrus JL Jr, Pippin J, Joseph A, Xu C, Blumenthal D, Tamayo A, Claypool K, McCourt D, Srikiatchatochorn A, Ford R. Identification of a cDNA for a human high molecular-weight B-cell growth factor. Proc Natl Acad Sci U S A. 1996;93(15):8154. doi:10.1073/pnas.93.15.8154-b. 8755619 PMC38894

[iyad031-B5] Ashburner M, Ball CA, Blake JA, Botstein D, Butler H, Cherry JM, Davis AP, Dolinski K, Dwight SS, Eppig JT, et al Gene ontology: tool for the unification of biology. The Gene Ontology Consortium. Nat Genet. 2000;25(1):25–29. doi:10.1038/75556. 10802651 PMC3037419

[iyad031-B6] Attrill H, Gaudet P, Huntley RP, Lovering RC, Engel SR, Poux S, Van Auken KM, Georghiou G, Chibucos MC, Berardini TZ, et al Annotation of gene product function from high-throughput studies using the Gene Ontology. Database. 2019;2019:baz007. doi:10.1093/database/baz007. 30715275 PMC6355445

[iyad031-B7] Banani SF, Lee HO, Hyman AA, Rosen MK. Biomolecular condensates: organizers of cellular biochemistry. Nat Rev Mol Cell Biol. 2017;18(5):285–298. doi:10.1038/nrm.2017.7. 28225081 PMC7434221

[iyad031-B8] Bansal P, Morgat A, Axelsen KB, Muthukrishnan V, Coudert E, Aimo L, Hyka-Nouspikel N, Gasteiger E, Kerhornou A, Neto TB, et al Rhea, the reaction knowledgebase in 2022. Nucleic Acids Res. 2022;50(D1):D693–D700. doi:10.1093/nar/gkab1016. 34755880 PMC8728268

[iyad031-B9] Basu S, Fey P, Jimenez-Morales D, Dodson RJ, Chisholm RL. Dictybase 2015: expanding data and annotations in a new software environment. Genesis. 2015;53:523–534. doi:10.1002/dvg.22867. 26088819 PMC4545684

[iyad031-B10] Bult CJ, Blake JA, Smith CL, Kadin JA, Richardson JE, Anagnostopoulos A, Asabor R, Baldarelli RM, Beal JS, Bello SM, et al Mouse Genome Database (MGD) 2019. Nucleic Acids Res. 2019;47(D1):D801–D806. doi:10.1093/nar/gky1056. 30407599 PMC6323923

[iyad031-B11] Cerqueira GC, Arnaud MB, Inglis DO, Skrzypek MS, Binkley G, Simison M, Miyasato SR, Binkley J, Orvis J, Shah P, et al The Aspergillus Genome Database: multispecies curation and incorporation of RNA-Seq data to improve structural gene annotations. Nucleic Acids Res. 2014;42(D1):D705–D710. doi:10.1093/nar/gkt1029. 24194595 PMC3965050

[iyad031-B12] Cooper L, Jaiswal P. The Plant Ontology: A Tool for Plant Genomics. Methods Mol Biol. 2016; 1374:89–114. doi:10.1007/978-1-4939-3167-5_5 26519402

[iyad031-B13] Costa M, Reeve S, Grumbling G, Osumi-Sutherland D. The Drosophila anatomy ontology. J Biomed Semantics. 2013; 4:32. doi:10.1186/2041-1480-4-32 24139062 PMC4015547

[iyad031-B14] Davis P, Zarowiecki M, Arnaboldi V, Becerra A, Cain S, Chan J, Chen WJ, Cho J, da Veiga Beltrame E, Diamantakis S, et al Wormbase in 2022-data, processes, and tools for analyzing Caenorhabditis elegans. Genetics. 2022;220(4):iyac003. doi:10.1093/genetics/iyac003. 35134929 PMC8982018

[iyad031-B15] Del Toro N, Shrivastava A, Ragueneau E, Meldal B, Combe C, Barrera E, Perfetto L, How K, Ratan P, Shirodkar G, et al The IntAct database: efficient access to fine-grained molecular interaction data. Nucleic Acids Res. 2022;50(D1):D648–D653. doi:10.1093/nar/gkab1006. 34761267 PMC8728211

[iyad031-B16] Diehl AD, Meehan TF, Bradford YM, Brush MH, Dahdul WM, Dougall DS, He Y, Osumi-Sutherland D, Ruttenberg A, Sarntivijai S, et al The Cell Ontology 2016: enhanced content, modularization, and ontology interoperability. J Biomed Semantics. 2016;7(1):44. doi:10.1186/s13326-016-0088-7. 27377652 PMC4932724

[iyad031-B17] Duck G, Nenadic G, Filannino M, Brass A, Robertson DL, Stevens R. A survey of bioinformatics database and software usage through mining the literature. PLoS One. 2016;11(6):e0157989. doi:10.1371/journal.pone.0157989. 27331905 PMC4917176

[iyad031-B18] Fabregat A, Jupe S, Matthews L, Sidiropoulos K, Gillespie M, Garapati P, Haw R, Jassal B, Korninger F, May B, et al The Reactome Pathway Knowledgebase. Nucleic Acids Res. 2018;46(D1):D649–D655. doi:10.1093/nar/gkx1132. 29145629 PMC5753187

[iyad031-B19] Fortriede JD, Pells TJ, Chu S, Chaturvedi P, Wang DZ, Fisher ME, James-Zorn C, Wang Y, Nenni MJ, Burns KA, et al Xenbase: deep integration of GEO & SRA RNA-seq and ChIP-seq data in a model organism database. Nucleic Acids Res. 2020;48:D776–D782. doi:10.1093/nar/gkz933. 31733057 PMC7145613

[iyad031-B20] Fungal-Anatomy-Ontology . A Structured Controlled Vocabulary for the Anatomy of Fungi. 2020. https://github.com/obophenotype/fungal-anatomy-ontology.

[iyad031-B21] Gaudet P, Livstone MS, Lewis SE, Thomas PD. Phylogenetic-based propagation of functional annotations within the Gene Ontology consortium. Brief Bioinform. 2011;12(5):449–462. doi:10.1093/bib/bbr042. 21873635 PMC3178059

[iyad031-B22] Gaudet P, Logie C, Lovering RC, Kuiper M, Lægreid A, Thomas PD. Gene Ontology representation for transcription factor functions. Biochim Biophys Acta Gene Regul Mech. 2021;1864(11–12):194752. doi:10.1016/j.bbagrm.2021.194752. 34461313

[iyad031-B23] Gene Ontology Consortium . The gene ontology in 2010: extensions and refinements. Nucleic Acids Res. 2010;38(suppl_1):D331–D335. doi:10.1093/nar/gkp1018. 19920128 PMC2808930

[iyad031-B24] Gill S, Gill R, Wicks D, Despotovski S, Liang D. Development of an HTS assay for Na+, K+-ATPase using nonradioactive rubidium ion uptake. Assay Drug Dev Technol. 2004;2(5):535–542. doi:10.1089/adt.2004.2.535. 15671651

[iyad031-B25] Gkoutos GV, Schofield PN, Hoehndorf R. The anatomy of phenotype ontologies: principles, properties and applications. Brief Bioinform. 2018;19(5):1008–1021. doi:10.1093/bib/bbx035. 28387809 PMC6169674

[iyad031-B26] Good BM, Van Auken K, Hill DP, Mi Huaiyu, Carbon Seth, Balhoff JP, Albou LP, Thomas PD, Mungall CJ, Blake JA, et al Reactome and the Gene Ontology: digital convergence of data resources. Bioinformatics. 2021;37(19):3343–3348. doi:10.1093/bioinformatics/btab325. 33964129 PMC8504636

[iyad031-B27] Haendel MA, Balhoff JP, Bastian FB, Blackburn DC, Blake JA, Bradford Y, Comte A, Dahdul WM, Dececchi TA, Druzinsky RE, et al Unification of multi-species vertebrate anatomy ontologies for comparative biology in Uberon. J Biomed Semantics. 2014;5(1):21. doi:10.1186/2041-1480-5-21. 25009735 PMC4089931

[iyad031-B28] Haendel MA, Neuhaus F, Osumi-Sutherland D, Mabee PM, Mejino JLV, Mungall CJ, Smith B. CARO—the common anatomy reference ontology. In: Burger A, Davidson D, Baldock R, editors. Anatomy Ontologies for Bioinformatics: Principles and Practice. London: Springer London; 2008. p. 327–349.

[iyad031-B29] Harris MA, Rutherford KM, Hayles J, Lock A, Bähler J, Oliver SG, Mata J, Wood V. Fission stories: using PomBase to understand *Schizosaccharomyces pombe* biology. Genetics 2022;220:iyab222. doi:10.1093/genetics/iyab222. 35100366 PMC9209812

[iyad031-B30] Hastings J, Owen G, Dekker A, Ennis M, Kale N, Muthukrishnan V, Turner S, Swainston N, Mendes P, Steinbeck C. ChEBI in 2016: improved services and an expanding collection of metabolites. Nucleic Acids Res 2016; 44(D1):D1214–9. doi:10.1093/nar/gkv1031. 26467479 PMC4702775

[iyad031-B31] Howe DG, Ramachandran S, Bradford YM, Fashena D, Toro S, Eagle A, Frazer K, Kalita P, Mani P, Martin R, et al The Zebrafish Information Network: major gene page and home page updates. Nucleic Acids Res. 2021;49(D1):D1058–D1064. doi:10.1093/nar/gkaa1010. 33170210 PMC7778988

[iyad031-B32] Kanehisa M, Furumichi M, Sato Y, Kawashima M, Ishiguro-Watanabe M. KEGG For taxonomy-based analysis of pathways and genomes. Nucleic Acids Res. 2022;51(D1):D587–D592. doi:10.1093/nar/gkac963. PMC9825424 36300620

[iyad031-B33] Keseler IM, Mackie A, Santos-Zavaleta A, Billington R, Bonavides-Martínez C, Caspi R, Fulcher C, Gama-Castro S, Kothari A, Krummenacker M, et al The EcoCyc database: reflecting new knowledge about Escherichia coli K-12. Nucleic Acids Res. 2017;45(D1):D543–D550. doi:10.1093/nar/gkw1003. 27899573 PMC5210515

[iyad031-B34] Kishore R, Arnaboldi V, Van Slyke CE, Chan J, Nash RS, Urbano JM, Dolan ME, Engel SR, Shimoyama M, Sternberg PW, et al Automated generation of gene summaries at the Alliance of Genome Resources. Database. 2020;2020:baaa037. doi:10.1093/database/baaa037. 32559296 PMC7304461

[iyad031-B35] Koopmans F, van Nierop P, Andres-Alonso M, Byrnes A, Cijsouw T, Coba MP, Cornelisse LN, Farrell RJ, Goldschmidt HL, Howrigan DP, et al SynGO: an evidence-based, expert-curated knowledge base for the synapse. Neuron. 2019;103(2):217–34.e4. doi:10.1016/j.neuron.2019.05.002. 31171447 PMC6764089

[iyad031-B36] Kuiper M, Bonello J, Fernández-Breis JT, Bucher P, Futschik ME, Gaudet P, Kulakovskiy IV, Licata L, Logie C, Lovering RC, et al The gene regulation knowledge commons: the action area of GREEKC. Biochim Biophys Acta Gene Regul Mech. 2022;1865(1):194768. doi:10.1016/j.bbagrm.2021.194768. 34757206

[iyad031-B37] Lamesch P, Berardini TZ, Li D, Swarbreck D, Wilks C, Sasidharan R, Muller R, Dreher K, Alexander DL, Garcia-Hernandez M, et al The Arabidopsis Information Resource (TAIR): improved gene annotation and new tools. Nucleic Acids Res. 2012;40(D1):D1202–D1210. doi:10.1093/nar/gkr1090. 22140109 PMC3245047

[iyad031-B38] Lang OW, Nash RS, Hellerstedt ST, Engel SR; SGD Project. An Introduction to the Saccharomyces Genome Database (SGD). Methods Mol Biol. 2018;1757:21–30. doi:10.1007/978-1-4939-7737-6_2. 29761454

[iyad031-B39] Lee R, Sternberg P. Building a cell and anatomy ontology of Caenorhabditis elegans. Comp Funct Genomics. 2003; 4:121–126. doi:10.1002/cfg.248 18629098 PMC2447384

[iyad031-B40] Lydeard JR, Lipkin-Moore Z, Sheu Y-J, Stillman B, Burgers PM, Haber JE. Break-induced replication requires all essential DNA replication factors except those specific for pre-RC assembly. Genes Dev. 2010;24(11):1133–1144. doi:10.1101/gad.1922610. 20516198 PMC2878651

[iyad031-B41] MacDougall A, Volynkin V, Saidi R, Poggioli D, Zellner H, Hatton-Ellis E, Joshi V, O’Donovan C, Orchard S, Auchincloss AH, et al Unirule: a unified rule resource for automatic annotation in the UniProt Knowledgebase. Bioinformatics. 2021;36(22–23):5562. doi:10.1093/bioinformatics/btaa663. 33821964 PMC8016456

[iyad031-B42] McDonald AG, Tipton KF. Fifty-five years of enzyme classification: advances and difficulties. FEBS J. 2014;281(2):583–592. doi:10.1111/febs.12530. 24103004

[iyad031-B43] McIntosh BK, Renfro DP, Knapp GS, Lairikyengbam CR, Liles NM, Niu L, Supak AM, Venkatraman A, Zweifel AE, Siegele DA, et al Ecoliwiki: a wiki-based community resource for Escherichia coli. Nucleic Acids Res. 2012;40(D1):D1270–7. doi:10.1093/nar/gkr880. 22064863 PMC3245172

[iyad031-B44] Meldal BHM, Bye-A-Jee H, Gajdoš L, Hammerová Z, Horáčková A, Melicher F, Perfetto L, Pokorný D, Lopez MR, Türková A, et al Complex Portal 2018: extended content and enhanced visualization tools for macromolecular complexes. Nucleic Acids Res 2019; 47(D1):D550–D558. doi:10.1093/nar/gky1001. 30357405 PMC6323931

[iyad031-B45] Mi H, Muruganujan A, Huang X, Ebert D, Mills C, Guo X, Thomas PD. Protocol update for large-scale genome and gene function analysis with the PANTHER classification system (v.14.0). Nat Protoc. 2019;14(3):703–721. doi:10.1038/s41596-019-0128-8. 30804569 PMC6519457

[iyad031-B46] Mungall CJ, Batchelor C, Eilbeck K. Evolution of the Sequence Ontology terms and relationships. J Biomed Inform. 2011;44(1):87–93. doi:10.1016/j.jbi.2010.03.002. 20226267 PMC3052763

[iyad031-B47] Mungall CJ, Torniai C, Gkoutos GV, Lewis SL, Haendel MA. Uberon, an integrative multi-species anatomy ontology. Genome Biol. 2012; 13:R5. doi:10.1186/gb-2012-13-1-r5 22293552 PMC3334586

[iyad031-B48] Munoz-Torres M, Carbon S. Get GO! Retrieving GO data using AmiGO, QuickGO, API, files, and tools. Methods Mol Biol. 2017;1446:149–160. doi:10.1007/978-1-4939-3743-1_11. 27812941

[iyad031-B49] Nadendla S, Jackson R, Munro J, Quaglia F, Mészáros B, Olley D, Hobbs ET, Goralski SM, Chibucos M, Mungall CJ, et al ECO: the Evidence and Conclusion Ontology, an update for 2022. Nucleic Acids Res. 2022;50(D1):D1515–D1521. doi:10.1093/nar/gkab1025. 34986598 PMC8728134

[iyad031-B50] Natale DA, Arighi CN, Blake JA, Bona J, Chen C, Chen SC, Christie KR, Cowart J, D'Eustachio P, Diehl AD, et al Protein Ontology (PRO): enhancing and scaling up the representation of protein entities. Nucleic Acids Res. 2017;45(D1):D339–D346. doi:10.1093/nar/gkw1075. 27899649 PMC5210558

[iyad031-B51] Paysan-Lafosse T, Blum M, Chuguransky S, Grego T, Pinto BL, Salazar GA, Bileschi ML, Bork P, Bridge A, Colwell L, et al Interpro in 2022. Nucleic Acids Res. 2022;51(D1):D418–D427. doi:10.1093/nar/gkac993. PMC9825450 36350672

[iyad031-B52] Quaglia F, Mészáros B, Salladini E, Hatos A, Pancsa R, Chemes LB, Pajkos M, Lazar T, Peña-Díaz S, Santos J, et al Disprot in 2022: improved quality and accessibility of protein intrinsic disorder annotation. Nucleic Acids Res. 2022;50(D1):D480–D487. doi:10.1093/nar/gkab1082. 34850135 PMC8728214

[iyad031-B53] Radivojac P, Clark WT, Oron TR, Schnoes AM, Wittkop T, Sokolov A, Graim K, Funk C, Verspoor K, Ben-Hur A, et al A large-scale evaluation of computational protein function prediction. Nat Methods. 2013;10(3):221–227. doi:10.1038/nmeth.2340. 23353650 PMC3584181

[iyad031-B54] Ramsey J, McIntosh B, Renfro D, Aleksander SA, LaBonte S, Ross C, Zweifel AE, Liles N, Farrar S, Gill JJ, et al Crowdsourcing biocuration: The Community Assessment of Community Annotation with Ontologies (CACAO). PLoS Comput Biol. 2021;17(10):e1009463. doi:10.1371/journal.pcbi.1009463. 34710081 PMC8553046

[iyad031-B55] Saverimuttu SCC, Kramarz B, Rodríguez-López M, Garmiri P, Attrill H, Thurlow KE, Makris M, de Miranda Pinheiro S, Orchard S, Lovering RC. Gene Ontology curation of the blood-brain barrier to improve the analysis of Alzheimer's And other neurological diseases. Database. 2021;2021:baab067. doi:10.1093/database/baab067. 34697638 PMC8546235

[iyad031-B56] Sayers EW, Beck J, Brister JR, Bolton EE, Canese K, Comeau DC, Funk K, Ketter A, Kim S, Kimchi A, et al Database resources of the National Center for Biotechnology Information. Nucleic Acids Res. 2020;48(D1):D9–16. doi:10.1093/nar/gkz899. 31602479 PMC6943063

[iyad031-B57] Sian L, Agapite J, Attrill H, Calvi BR, Crosby MA, dos Santos G, Goodman JL, Goutte-Gattat D, Jenkins VK, Kaufman T, et al Flybase: a guided tour of highlighted features. Genetics. 2022;220:iyac035. doi:10.1093/genetics/iyac035. 35266522 PMC8982030

[iyad031-B58] Skrzypek MS, Binkley J, Binkley G, Miyasato SR, Simison M, Sherlock G. The Candida Genome Database (CGD): incorporation of Assembly 22, systematic identifiers and visualization of high throughput sequencing data. Nucleic Acids Res. 2017;45(D1):D592–D596. doi:10.1093/nar/gkw924. 27738138 PMC5210628

[iyad031-B59] Smith B, Ceusters W, Klagges B, Köhler J, Kumar A, Lomax J, Mungall C, Neuhaus F, Rector AL, Rosse C. Relations in biomedical ontologies. Genome Biol. 2005;6(5):R46. doi:10.1186/gb-2005-6-5-r46. 15892874 PMC1175958

[iyad031-B60] Smith JR, Hayman GT, Wang S-J, Laulederkind SJF, Hoffman MJ, Kaldunski ML, Tutaj M, Thota J, Nalabolu HS, Ellanki SLR, et al The Year of the Rat: The Rat Genome Database at 20: a multi-species knowledgebase and analysis platform. Nucleic Acids Res. 2020;48:D731–D742. doi:10.1093/nar/gkz1041. 31713623 PMC7145519

[iyad031-B61] Thomas PD . The Gene Ontology and the meaning of biological function. Methods Mol Biol. 2017;1446:15–24. doi:10.1007/978-1-4939-3743-1_2. 27812932 PMC6438694

[iyad031-B62] Thomas PD, Ebert D, Muruganujan A, Mushayahama T, Albou LP, Mi H. PANTHER: making genome-scale phylogenetics accessible to all. Protein Sci. 2022;31(1):8–22. doi:10.1002/pro.4218. 34717010 PMC8740835

[iyad031-B63] Thomas PD, Hill DP, Mi H, Osumi-Sutherland D, Van Auken K, Carbon S, Balhoff JP, Albou L-P, Good B, Gaudet P, et al Gene Ontology Causal Activity Modeling (GO-CAM) moves beyond GO annotations to structured descriptions of biological functions and systems. Nat Genet. 2019;51(10):1429–1433. doi:10.1038/s41588-019-0500-1. 31548717 PMC7012280

[iyad031-B64] Thompson W, Zuk RT. Acylation of CDP-monoacylglycerol cannot be confirmed. J Biol Chem. 1983;258(16):9623. doi:10.1016/S0021-9258(17)44541-8. 6885763

[iyad031-B65] Torto-Alalibo T, Collmer CW, Gwinn-Giglio M. The Plant-Associated Microbe Gene Ontology (PAMGO) Consortium: community development of new Gene Ontology terms describing biological processes involved in microbe-host interactions. BMC Microbiol. 2009;9 Suppl1:S1. doi:10.1186/1471-2180-9-S1-S1 19278549 PMC2654661

[iyad031-B66] The UniProt Consortium . Uniprot: the universal protein knowledgebase. Nucleic Acids Res. 2017;45(D1):D158–D169. doi:10.1093/nar/gkw1099. 27899622 PMC5210571

[iyad031-B67] Walls RL, Cooper L, Elser J, Gandolfo MA, Mungall CJ, Smith B, Stevenson DW, Jaiswal P. The Plant Ontology facilitates comparisons of plant development stages across species. Front Plant Sci. 2019;10:631. doi:10.3389/fpls.2019.00631. 31214208 PMC6558174

[iyad031-B68] Winsor GL, Lo R, Ho Sui SJ, Ung KS, Huang S, Cheng D, Ching WK, Hancock RE, Brinkman FS. Pseudomonas aeruginosa Genome Database and PseudoCAP: facilitating community-based, continually updated, genome annotation. Nucleic Acids Res. 2005;33(Database issue):D338–D343. doi:10.1093/nar/gki047. 15608211 PMC540001

